# The regulation of antiviral activity of interferon epsilon

**DOI:** 10.3389/fmicb.2022.1006481

**Published:** 2022-10-25

**Authors:** Fu-Rong Zhao, Wei Wang, Qin Zheng, Yan-Ge Zhang, Jianming Chen

**Affiliations:** ^1^Fujian Key Laboratory on Conservation and Sustainable Utilization of Marine Biodiversity, Fuzhou Institute of Oceanography, Minjiang University, Fuzhou, China; ^2^Fujian Provincial Key Laboratory for Prevention and Control of Animal Infectious Diseases and Biotechnology, Longyan University, Longyan, China

**Keywords:** interferon epsilon, antiviral, viral interference, biological activities, type I IFNs

## Abstract

Interferon epsilon (IFN-ε) is a type I IFN. Some biological properties has been identified in many species, such as antiproliferative, anti-tumor, and antiviral effects, of IFN-ε, which are much weaker than those of IFN-α, have also been revealed. It has been shown to play a role in mucosal immunity and bacterial infection and in the prevention of certain sexually transmitted diseases, such as human immunodeficiency virus (HIV). This paper reviews the known activity of IFN-ε, particularly in some viruses. In general, this review provides a better understanding of effective IFN-ε treatment in the future.

## Introduction

As the concept of viral interference was put forward ~60 years ago, the ability of viruses to infect cells has reduced. The elucidation of the biological characteristics of interferons (IFNs) has gained increasing scientific and clinical interest. IFNs can be divided into three categories according to their genetic, structural, and functional characteristics and their cell surface receptors: types I, II, and III. Type I IFNs are a family of pro-inflammatory cytokines produced by all nucleated cells. Their main purpose is to inhibit pathogenic infection (Tailor et al., 2006; McNab et al., 2015; Makris et al., 2017; Negishi et al., 2018). In humans, type I IFNs consist of 17 related proteins, including 13 distinct IFN-α, IFN-β, IFN-κ, IFN-ω, and IFN-ε (Pestka, 2007). Except for IFN-ω and IFN-κ, mice have the same IFN subtypes (Weissmann and Weber, 1986).

Interferon epsilon (IFN-ε) is a type I IFN discovered in 2004 (Hardy et al., 2004). Although it has low amino acid sequence homology (~30%) compared with IFN-α/β, it has an unusual expression profile and different regulation. Its expression is neither induced by the pattern recognition receptor pathway nor significantly stimulated by viruses, but is mediated by binding to IFN-α/β receptors (IFNAR1 and IFNAR2), and therefore is a member of type I IFNs (de Weerd et al., 2007; Day et al., 2008; Fung et al., 2013).

Interferon epsilon has been found to exist in several organisms, including humans, mice, pigs, cattle, *Ovis aries*, dogs, horses, rhesus macaques, pangolins, and fishes (Hardy et al., 2004; Day et al., 2008; Sun et al., 2009; Walker and Roberts, 2009; DeCarlo et al., 2010; Sang et al., 2010; Fischer et al., 2018; Fung et al., 2013; Hermant et al., 2013; Yang et al., 2013; Demers et al., 2014; Guo et al., 2015, 2020; Choo et al., 2016; Robertsen, 2018). Interestingly, unlike IFN-α and IFN-β, IFN-ε is constitutively expressed in various mucosal tissues, mainly the lungs, small intestine, and reproductive tissues, and is highly expressed in the female reproductive tract, especially in the uterus, cervix, vagina, and ovary (Day et al., 2008; DeCarlo et al., 2010; Fung et al., 2013; Hermant et al., 2013).

However, most current research focuses on IFN-α and IFN-β, and little is known about the other subtypes of IFN-ε. In addition, the biochemical and biological characteristics of IFN-ε remain to be elucidated. To further understand the function of IFN-ε, relevant information is summarized to provide a reference for future research.

## Molecular features

Interferon epsilon was first described in mice and humans in 2004. In mice, it is located at the extreme 3′ end of the mouse type I IFN locus. It has no intron, is transcribed toward the 5′ end of chromosome 4, and encodes an open reading frame of 192 amino acids, from which complementary DNA (cDNA) was successfully amplified using mouse embryonic fibroblast cells. In humans, the IFN-ε gene is located on chromosome 9 (9p21) of the type I IFN locus (Dandoy et al., 1984; Cahilly et al., 1985; Kelley and Pitha, 1985; van der Korput et al., 1985). It encodes an open reading frame of 208 amino acids from which cDNA was first amplified in human WISH cells (Hardy et al., 2004). The main characteristics of IFN-ε in humans and mice are listed in Table 1. The analysis of their amino acid sequences showed that they had 75% sequence homology (54% identity). In addition, two cysteine residues (at positions 32 and 142 of the mature sequence) were predicted to form critical disulfide in type I IFNs and were constant in them (Marks et al., 2019).

**Table 1:** The main characteristics of interferon epsilon (IFN-ε) from humans and mice.

**Characteristics**	**Human**	**Mouse**
IFN type	Type I	Type I
Gene structure	The mature 185-amino-acid and 15 residues. The secondary structure of the human IFN-ε1 has five putative α helices. Helix F on human IFN-ε1 was located on its C-terminal extension and is not present on mouse IFN-ε1	The mature 185-amino-acid secondary structure of the mouse IFN-ε1 has five putative α helices
Amino acid	They share 54% amino acid identity plus 15% amino acid similarity	
Number of subtypes	1	1
Pseudogenes	IFN-ε1	IFN-ε1
Length of mature peptides	187	187
Chromosome locus	Chromosome9	Chromosome4
Tissue distribution	Highly expressed in the placenta, constitutively expressed in the brain, coronary smooth muscle endothelial cells, microvascular endothelial cells, and bronchial epithelial cells	Expressed in the ovaries, uterus, fibroblasts, lung, brain, adrenal glands, and heart but was not detected in thymus, spleen, testis, liver, and kidney
Cell	The human prostate cancer cell line PC-3, amnion-derived WISH cells, SK-MEL28 melanoma cells, and Daudi cells and very weakly in MCF-7 human breast cancer cells	L929, MEFs, and M1 cell

## Regulation of IFN-ε expression

Interferon epsilon has a signature similar to that of type I IFNs in stimulating immune mediators and signaling pathways. For example, IFN-ε can activate the JAK-STAT signaling pathway and upregulate expression levels of PKR, MxA, IRF7, ISG15, and 2′5′OAS (Hardy et al., 2004; Day et al., 2008; Yang et al., 2013). IFN-ε levels are also upregulated following simulation of tumor necrosis factor α (TNF-α), which is involved in the stabilization and translation mechanism of IFN-ε messenger RNA (mRNA) in the absence of transcriptional activation. As a result, the engagement of type I IFNR was induced, upregulating the expression and activation of STAT1 and increasing the expression of RIG-I (Matsumiya et al., 2007). However, unlike other identified type I IFN genes that could be activated by pattern recognition receptor pathways, IFN-ε was not transcriptionally upregulated in primary bone marrow-derived macrophages, murine embryonic fibroblasts, and murine macrophage cell lines following treatment with synthetic ligands (Fung et al., 2013).

In general, gene expression is regulated by gene replication, transcription, mRNA splicing, stability and translation, protein post-translational modification and stability, and other factors (Day and Tuite, 1998). The full-length 5′ untranslated regions of the IFN-ε mRNA sequences, including two stable stem loop structures (loops 1 and 2), markedly suppressed constitutive expression of IFN-ε mRNA (Matsumiya et al., 2013). However, only loop 1 is essential to enhance mRNA expression when the loop structure is disrupted. Interestingly, a molecular transporter and chaperone (IPO9) binds to the stem-loop structures of IFN-ε 5′UTR. This could affect constitutive expression of IFN-ε. IFN-ε decreases following IPO9 overexpression and increases in response to IPO9 silencing (Matsumiya et al., 2013). These results indicate that IPO9 is a negative and specific post-transcriptional regulator of IFN-ε mRNA expression that participates in stem-loop structure 1.

Interferon epsilon can also be modulated by hormones and seminal plasma (Aumüller and Riva, 1992; Fung et al., 2013). In a previous study, IFN-ε varied about 30-fold at different stages of the estrous cycle. While the lowest levels of IFN-ε mRNA were detected during diestrus, the highest levels were in estrus, and IFN-ε was induced over 6-fold following estrogen administration (Fung et al., 2013). However, no changes in IFN-α and IFN-β expression were found, suggesting that IFN-ε expression could be regulated by hormones. Previous studies have revealed that a progesterone receptor response element exists in the promoter region of the IFN-ε gene (Hardy et al., 2004). In addition, seminal plasma, an important transport medium for spermatozoa that traverse the female cervix and uterus after coitus, has been reported to upregulate IFN-ε expression in cervical and vaginal tissues (Aumüller and Riva, 1992; Sharkey et al., 2007). These results suggest that IFN-ε is regulated by hormones. The expression of the IFN-ε gene in different tissues and cell types is listed in Table 2.

**Table 2:** IFN-ε gene expression in different tissues and cell types.

	**IFN-ε**	**IFN-α**	**IFN-β**	**References**
Species	Human, mice, swine, cattle, dog, rhesus macaques, pangolin, fish, Ovis aries	Almost all animals	Almost all animals	Hardy et al., 2004; Day et al., 2008; Sun et al., 2009; DeCarlo et al., 2010; Sang et al., 2010; Fung et al., 2013; Hermant et al., 2013; Demers et al., 2014; Guo et al., 2015; Choo et al., 2016; Robertsen, 2018
Subtype	Only one number has been identified in these species	13, 6, 17, 14, and 9 numbers in human, horse, pig, cattle, and dog, respectively	1, 4, and 1 number in human, horse, and pig, respectively	Pestka, 2007; Fischer et al., 2018
Amino acid	Only 30% amino acid homology to a consensus IFN-α sequence and IFN-β			Hardy et al., 2004; Li et al., 2018
Receptors	IFNAR-1/IFNAR2	IFNAR-1/IFNAR2	IFNAR-1/IFNAR2	de Weerd et al., 2007; Day et al., 2008; Fung et al., 2013
Gene locus	9p21	9p21	9p21	Dandoy et al., 1984; Cahilly et al., 1985; Kelley and Pitha, 1985; van der Korput et al., 1985
Expression pattern	Constitutively expressed	Ubiquitously expressed	Ubiquitously expressed	Day et al., 2008; DeCarlo et al., 2010; Fung et al., 2013; Hermant et al., 2013

## Biological properties of IFN-ε

Similar to other types of IFN, IFN-ε has been shown to have antiviral activity *in vitro* and *in vivo*. For example, VV-HIV-IFN-ε, a recombinant vaccine virus (VV) co-expressing the human immunodeficiency virus (HIV) gag or pol gene and murine IFN-ε, was found to decrease the growth of VV in L929 murine cell lines and improve its ability to upregulate activation markers (CD69 and CD86) and antiviral protein expression (Day et al., 2008). However, there are several differences between IFN-ε and other IFNs. First, the antiviral activity of IFN-ε is much weaker than that of IFN-α and IFN-β. In a study by Peng et al. (2007), amnion-derived WISH cells infected with vesicular stomatitis virus (VSV) were used, and compared with IFN-α-2b, IFN-ε has less antiviral, natural killer cell cytotoxicity, and antiproliferative activities. In addition, the recombinant canine IFN-α gene (rCaIFN-α7) also exhibited ~16-fold greater potential than rCaIFN-ε in promoting natural killer cell cytotoxicity (Peng et al., 2007). Second, IFN-ε has broader cross-species activity than rCaIFN-α. It can also exhibit certain antiviral activity in cells derived from species that have nearly relative expectations for homologous cells. For example, rCaIFN-α is approximately three times more active than rCaIFN-ε in canine cells (e.g., MDCK); however, rCaIFN-α showed inferior biological properties than rCaIFN-ε in heterologous cells, such as MDBK, CRFK, and Wish (Yang et al., 2013). In addition, BoIFN-ε has no protective effect on MDCK and BHK-21 cells after VSV challenge, but its biological effect can be detected in MDBK, BT, EBK, and PK-15 cells (Guo et al., 2015). Third, IFN-ε also differs from IFN-α in macrophages by inducing an antiviral state mediated by different factors. Some studies have revealed that the amounts or levels of genes induced by type I IFN, IL-6, and TNF pathways in response to IFN-α and IFN-ε are not identical despite some of them (IL-1α, IL-1RA, IL-4, VEFG, and GCSF) overlapped (Tasker et al., 2016). IFN-α mediates and upregulates more genes than IFN-ε in the type I IFN signaling pathway, whereas IFN-ε induces more genes in the TNF-α pathway, ROS generation, and phagocyte activation than IFN-α, which blocks the replication of HIV (Tasker et al., 2016). Except for its antiviral effects, IFN-ε has a signature similar to that of type I IFNs in exhibiting an apparent antiproliferative and antitumor effect. It can significantly inhibit the growth of A72 canine tumor cells and MDCK canine epithelial cells in a dose-dependent manner (Yang et al., 2013).

Interferon epsilon could also be constitutively expressed in the lungs and in an allergic BALB/c model, and IFN-ε mRNA levels were upregulated compared with those in healthy mice (Day et al., 2008), suggesting that it could play a role in mucosal immunity. To further investigate whether IFN-ε could induce mucosal protective immunity, IFN-ε infection with VV-HIV-IFN-ε was used to evaluate the role of IFN-ε in mucosal immunity. Xi et al. (2012) found that VV-HIV-IFN-ε induced rapid VV clearance, and VV-specific CD8+CD107a+ IFN-γ+ levels were increased in the lungs. In addition, this infection elevated lymphocyte homing to lung alveoli with decreased inflammation and CD3 ^high^ CCR7 ^high^ CD62L ^low^ in lung lymph nodes (Xi et al., 2012). Interestingly, IFN-ε enhanced HIV-specific effects. However, no memory CD8+T cell responses were detected in the Peyer's patches, spleen, and genito-rectal nodes when IFN-ε was used in a heterologous intranasal HIV-1 prime-boost vaccination regimen. Additionally, it was found that unlike other type I IFNs, IFN-ε could promote the migration of antigen-specific CD8+ T cells to the gut according to the results of analyzing the homing markers α4β7 and CCR9 (Xi et al., 2012). The latest research shows that it also has similar constitutive expression in the human female reproductive tract (hFRT), and it has a crucial role in regulating protective immunity (Bourke et al., 2022).

However, the IFN-ε protein used in these studies was mostly expressed in the form of an inclusion body. It requires a lot of time and complex procedures to be correctly folded into a functional protein that represents the native conformation. Therefore, the soluble protein can stimulate studies to explore its biological properties and the mechanism of response to viral infection.

## Positive response to viruses or diseases

### Vitiligo and intracerebral hemorrhage

As described above, IFN-ε can be constitutively expressed in the brain (Peng et al., 2007); therefore, it is involved in maintaining the structure and function of the brain. Previous studies have shown that IFN-ε-mediated hyaluronic acid-mediated motility receptor is involved in the formation of brain microvessels (Lokeshwar and Selzer, 2000). This suggests that they may play a role in immune-associated diseases in humans. Vitiligo is a common skin disorder in which melanocytes in the skin disappear. Recently, inflammation modulated by IFN-ε was shown to be related to the pathophysiology of vitiligo. In a study by Cho et al. (2013), the relationship between the non-sense polymorphism (rs2039381 and Gln71Stop) of IFN-ε and susceptibility to vitiligo was investigated. They found no apparent differences in the rs2039381 (Gln71Stop) of IFN-ε between patients with non-segmental vitiligo and healthy humans. However, there was a significant difference in the distribution of IFN-ε non-sense polymorphism (rs2039381 and Gln71Stop) between patients with non-segmental vitiligo (age < 18-year-old) and the control group. This study revealed that the rs2039381 (Gln71Stop) polymorphism of IFN-ε might be correlated with the early stages of vitiligo in patients with non-segmental vitiligo. In lateral studies (Kim et al., 2014), the non-sense SNP (rs2039381 and Gln71Stop) of IFN-ε was also selected to identify a correlation between a non-sense polymorphism of IFN-ε and ischemic stroke or intracerebral hemorrhage stroke (two main subtypes of stroke that threaten public health severity) in Korean people. Kim et al. (2014) showed that it was also related to intracerebral hemorrhage stroke in the Korean population, according to evidence that the T allele frequency of rs2039381 was significantly higher in humans suffering from intracerebral hemorrhage stroke than in healthy individuals.

### Human immunodeficiency virus

Interferon epsilon is constitutively expressed in mucosal tissues, such as the lungs, small intestine, and reproductive tissues, whereas it is highly expressed in the female genital mucosa (Hardy et al., 2004; Fung et al., 2013). TNF-α induces IFN-ε, but not IFN-α/β, in HeLa cells (Matsumiya et al., 2007). This suggests that it may play an important role in some reproductive diseases, such as HIV and cervical cancer (CC). It can regulate mucosal immunity, resist viral and bacterial infections, and inhibit HIV replication. Its expression is negatively correlated with progesterone levels (Li et al., 2018).

Human immunodeficiency virus has been an important factor causing acquired immunodeficiency syndrome (AIDS) for 300 years (Mamo et al., 2010). Since its discovery, HIV/AIDS has been responsible for the deaths of more than 25 million individuals worldwide, and more than 2 million individuals acquire HIV infection every year. Among the factors leading to this infection, sexual transmission is the major route that disproportionately affects women (Date and Destache, 2013). Increasing evidence has shown that IFN-ε could significantly prevent the transmission of HIV (Vishwanathan et al., 2011; Curlin et al., 2013). For example, increasing levels of the IFN-*ε* gene and its protein expression in the cervical epithelium are related to reduced expression of genes involved in HIV-1 integration and replication (Abdulhaqq et al., 2016). In addition, susceptibility to HIV infection was negatively related to IFN-ε levels during the menstrual cycle, suggesting that this cytokine is likely conducive to anti-HIV responses. Several mechanisms have been proposed. IFN-ε plays a role in the early stages of the HIV life cycle (from viral entry to nuclear import) and blocks HIV-1 replication by inducing CC-chemokines, downregulating CCR5, and inhibiting reverse transcription and nuclear import; this effect on macrophages was maximal after 24 h of treatment and was reversible (Tasker et al., 2016). Interestingly, IFN-ε showed little or no significant difference in the protective effect of activated CD4+ T cells and transformed cell lines, except activated CD4+ T cells, which were infected with replication-competent HIV-1 at a low multiplicity of infection (MOI). However, this protection did not appear to operate through APOBEC3A and SAMHD1 (Tasker et al., 2016). Further efforts were made by Garcia-Minambres et al. (2017) to reveal the anti-HIV activities of IFN-ε *in vitro*; its antiviral activity is mediated at the different stages of the HIV replication cycle by inducing the expression of HIV restriction factors TRIM5α, MX2, HERC5, BST2, IFITM3, and APOBEC3G, and this induction was comparable to that of IFN-α and IFN-β. In this study, recombinant IFN-ε impaired HIV infection from HIV entry to the translation of viral proteins. IFN-ε also remarkably reduces the infectivity of progeny virion particles at that stage of infection by inducing the expression of HIV restriction factors, such as IFITM3 (Garcia-Minambres et al., 2017). Overall, these findings have unveiled new insights into the innate immune response to HIV infection, making it possible to develop new, safer HIV prevention strategies to suppress the transmission of HIV in women.

### Cervical cancer

Cancer is a challenging global problem. Research has shown that the expression of IFN-ε mRNA can represent a potential molecular marker for CC. It may play an important role in intracellular homeostasis and affect immune-related events in cervical carcinogenesis. In addition, the expression of IFN-ε was not related to HPV infection or genotype, and the possible variation in copy number did not affect its expression in CC.

### Zika virus

In 1947, the Zika virus, a mosquito-borne disease transmissible from human to human through the bites of Aedes species, was named after the Zika Forest area of Uganda, in which it was first isolated from the serum of a pyrexial rhesus monkey (D'Ortenzio et al., 2016; Maharajan et al., 2016). Similar to HIV, Zika virus is a sexually transmitted pathogen that is vulnerable to IFITM-mediated restriction, indicating that IFN-ε may also contribute to inhibiting the spread of Zika virus (Fung et al., 2013; Savidis et al., 2016).

### Herpes simplex virus 2

Herpes simplex virus 2, a member of the herpesvirus family, is a sexually transmitted infection. IFN-ε-deficient mice were used in a study by Fung et al. (2013). Compared with the control group, mice had remarkably more severe epidermal lesions on days 6 and 7 after being challenged with a sublethal dose of a clinical isolate of herpes simplex virus 2 (HSV-2) strain 186. Viral titers in infected vaginal tissues of experimental mice were increased on day 3 post-infection compared with wild-type animals; at 7 days post-infection, IFN-ε-deficient mice had significantly higher viral levels in the spinal cord and brain stem.

### *Chlamydia muridarum*  

*Chlamydia muridarum*, a Gram-negative obligate intracellular pathogen, is an important causative agent of sexually transmitted infections worldwide. IFN-ε-deficient mice challenged with *C. muridarum* at a sublethal dose of 5 × 10^4^ IFU intravaginally showed that mice showed more severe clinical signs of disease from 7 to 30 days after infection. Interestingly, in this study, more bacteria were detected in the vaginal swabs of IFN-ε-deficient mice after infection (Fung et al., 2013). This suggests that IFN-ε may also be beneficial for the control of certain bacterial infections. Compared with IFN-α family members, IFN-ε exerts lower antiviral, antiproliferative, and natural cell killer-enhancing activities (Xi et al., 2012).

### SARS-CoV-2

SARS-CoV-2 (COVID-19) was first reported in Wuhan, China, and causes severe respiratory diseases such as pneumonia and lung failure. It has spread in China and the world, reaching a pandemic level (Ahn et al., 2020). According to epidemiological investigations, the mortality rate of men seems to be higher than that of women (Grasselli et al., 2020; Guan et al., 2020; Livingston and Bucher, 2020), which may be closely related to the characteristics of IFN-ε, confirming Fung's theory (Fung et al., 2013; Afsar and Afsar, 2020). In women, IFN-ε can only act in the reproductive tract, but not in other organs. This may be why the mortality rate of women was lower than that of men. Recent studies have shown pre-activated innate immunity in children leads to a more effective anti-COVID-19 response after infection (Pierangeli et al., 2022). The basal transcription of type I/III IFN gene in COVID-19 infected and non-infected was the highest in children and decreased with age. Amon people infected with COVID-19, only IFN-ε and IFN-ω levels were significantly higher in children and young adults, which also explained that IFN-ε played an important role in the fight against COVID-19.

## Silent to other viral infections or diseases

### Simian immunodeficiency virus

Similarity to HIV, simian immunodeficiency virus (SIV) invades host cells and makes full use of cells for transcription *in vitro*, this mechanism is considered to be the culprit of HIV/AIDS virus type 1 in the world (Sakuma and Takeuchi, 2012). Some studies revealed that no significant difference was detected in the expression of IFN-ε between healthy and acutely infected SIV rectal tissues or at 10, 14, or 28 days post-infection by immunofluorescence staining and quantitative real-time polymerase chain reaction (RT-PCR), indicating that acute SIV infection of the rectal tissue did not change the expression of IFN-ε mRNA (Demers et al., 2014).

### *Mycobacterium tuberculosis*  

Tuberculosis (TB), a zoonotic disease in primates, is caused by *M. tuberculosis*. Rhesus and cynomolgus are also good animal models for studying TB in humans because they show clinical symptoms and histopathologic lesions similar to those of humans infected with TB (Walsh et al., 1996; Capuano et al., 2003). Roodgar et al. (2015) reported that there was no relationship between the levels of IFN-ε mRNA expression in peripheral blood mononuclear cells (PBMCs) and the severity of TB lesions in the lungs of rhesus macaques in pre-infection and various stages of post-infection after animals were infected with *M. tuberculosis*.

### Theiler's murine encephalomyelitis virus

Theiler's murine encephalomyelitis virus (TMEV) is a small RNA picornavirus widely used as a model to study the initiation and progression of multiple sclerosis, an inflammatory demyelinating disease of the central nervous system (Johnson et al., 2014). KJ07, a derivative of TMEV expressing eGFP, was used to infect cells transduced with lentiviral bicistronic vectors co-expressing the fluorescent protein mCherry and murine IFN-αA (pPH50) or IFN-ε (pPH49). The antiviral activity of IFN-ε in the transduced cells was detected at a lower level than that of IFN-αA by fluorescence-activated cell sorting (FACS) analysis. However, compared with untransduced mCherry-negative cells with the same population, no significant difference was observed in protecting cells expressing IFN-ε against viral infection (Hermant et al., 2013). Thus, intracellular expression of IFN-ε does not trigger resistance to viral infection.

### Mengovirus

Mengovirus is a single-stranded RNA virus of the genus Cardiovirus and family Picornaviridae. It often causes severe systemic and lethal infections in mice. In an analysis of quantitative RT-PCR, Hermant et al. (2013) investigated changes in IFN-ε expression levels in mice after infection with Mengovirus and found that the spinal cord, heart, brain, and other organs were the most infected 4 days after intraperitoneal injection of Mengovirus. Moreover, the expression of IFN-β was upregulated, but the expression of IFN-ε was not.

### Pre-term birth

Pre-mature birth is a serious public health problem as it is the main cause of infant death (Walani, 2020). Studies have shown that IFN-ε has no significant correlation with intrauterine infection (chorioamnionitis) or pre-eclampsia, resulting in pre-term birth. Medical observations have shown that the probability of pre-mature births is increasing. This may be because IFN-ε in women with pre-term pre-eclampsia increases the risk of pre-mature births (Taylor et al., 2022). Therefore, improving the understanding of type I IFN during pregnancy will contribute to the development of this field and clarify the immunological mechanisms that lead to disease complications.

## Conclusions and future perspectives

Modern studies on the induction and biological activity of IFN-ε have improved our understanding of type I IFN family members and made important contributions to promoting their application. As mentioned earlier, IFN-ε has been demonstrated to play a role in mucosal immunity against viral and bacterial infections and some types of sexually transmitted diseases; therefore, it has great potential as a novel therapeutic to control immunodeficiency-related diseases or reproductive infections, such as HIV and Zika virus, and to regulate immunity in a tissue-specific manner.

Interferon epsilon is a unique type I IFN that has a distinct role in viral infection and disease manifestation compared with IFN-α/β. IFN-ε showed weaker antiviral activity compared with IFN-α/β, which is important but overlooked in this field. It will be beneficial to the field if the authors summarize the assay system and antiviral 50% growth inhibition concentration (IC_50_) of IFN-ε and discuss the cell-type-dependent activity of IFN-ε.

Type 1 IFNs induce antiviral activity by inhibiting viral gene transcription. The IC_50_ was calculated to assess the antiviral activity of IFN-ε. Generally, it is possible that the bioassay commonly used to assess the antiviral function of type I IFNs is not appropriate for unique IFNs. For example, the difference between the studies by Tasker et al. and Abdulhaqq et al. regarding the anti-HIV activity of IFN-ε in transformed cell lines. Abdulhaqq et al. used international units to normalize protein concentrations of IFN-ε and IFN-α/β, in which the concentration of IFN-ε may be significantly higher than that of IFN-α/β due to the known weak antiviral activity of IFN-ε in the bioassay. Thus, although IFN-α, IFN-β, and IFN-ε bind to common type 1 receptors, IFNaR1 and IFNaR2, their binding and biological activities exhibit species-, tissue-, and cell-specific differences, probably due to three-dimensional conformational differences among type 1 IFN ligands (Roberts et al., 1997).

Previous studies have shown that higher induction of IFN-α and IFN-β causes negative host side effects, such as elevated persistence (Bosinger et al., 2009; Boasso et al., 2011; Wijesundara et al., 2014). Therefore, it is important to develop effective therapeutic and vaccine strategies for devastating diseases in humans and other animals; however, it is not known whether IFN-ε can be detrimental to humans or other animals when used for treatment or vaccination. Further studies are needed to address these important questions. In addition, although IFN-ε displayed antiviral activity, this effect is complex. For example, it can display positive activities in response to HSV-2 and *C. muridarum*, but not to SIV, TB, TMEV, and Mengovirus. Recent studies have also shown that IFN-ε with cetacean-specific mutations in mucosal immunity-related genes plays an important role in protecting and/or regulating immune responses against viruses (Chung et al., 2022). However, abundant evidence indicates the importance of IFN-ε in mucosal immunity, and tissue sampling may be important in assessing IFN-ε expression. Thus, more studies are needed to identify IFN-ε in more diverse species and to evaluate its biological characteristics in other viral and bacterial infections. Based on these research foundations, it is conducive to promoting its application.

## Author contributions

F-RZ, WW, QZ, and Y-GZ wrote the draft of this manuscript. JC gave instructions and proofread the article. All authors contributed to the article and approved the submitted version.

## Funding

This work was supported by the Natural Science Foundation of Fujian Province of China (2021J011044) and the Fujian Provincial Key Laboratory for Prevention and Control of Animal Infectious Diseases and Biotechnology (ZDSYS2020002).

## Conflict of interest

The authors declare that the research was conducted in the absence of any commercial or financial relationships that could be construed as a potential conflict of interest.

## Publisher's note

All claims expressed in this article are solely those of the authors and do not necessarily represent those of their affiliated organizations, or those of the publisher, the editors and the reviewers. Any product that may be evaluated in this article, or claim that may be made by its manufacturer, is not guaranteed or endorsed by the publisher.
